# Risk, Technology, and Moral Emotions: Reply to Critics

**DOI:** 10.1007/s11948-020-00196-3

**Published:** 2020-02-18

**Authors:** Sabine Roeser

**Affiliations:** Faculty of Technology, Policy and Management, TU Delft, Delft, The Netherlands

## Introduction

First of all, I would like to thank Colleen Murphy and Neelke Doorn for each having organized a book symposium about my book at conferences, and I would like to thank the commentators at these sessions for taking the time to study my book and to provide comments. I specifically thank the authors of the written comments that are included in this book symposium: Madeleine Hayenhjelm, Sven Nyholm and Steffen Steinert. It is truly an honor to have colleagues who have thoroughly read my book, shed their critical light on it, challenged me to explain ideas further and explore relevant, unchartered territory in more detail. In what follows, I will discuss the commentaries and respond to issues that may need further clarification or development.

## Reply to Hayenhjelm

I would like to thank Madeleine Hayenhjelm for her nice summary of my ideas and of her detailed discussion of some of my key claims. Hayenhjelm focuses primarily on my metaethical arguments developed in chapter 5, where I present an alternative theory of emotions to the standard approaches to emotions in risk research. Rather than seeing emotion as opposed to rationality, as in the common framework of Dual Process Theory, my approach is grounded in a cognitive theory of emotions. I argue that emotions can be cognitive and affective at the same time, and that they can provide us with important moral insights. I argue that emotions are not opposed to rationality, rather, they are a form and source of *practical* rationality. Hayenhjelm agrees with some of my arguments but not with all of them. In what follows I will discuss three main points of contention that Hayenhjelm has raised against my theory, related to the issue of concrete cases versus general principles (“Responses to concrete cases versus general principles” section), criteria of rightness of emotions (“Reliability of emotions and criteria for correctness” section) and inclusion of laypeople (“The role of laypeople” section).

### Responses to Concrete Cases Versus General Principles

Hayenhjelm critically engages with my claim that emotions are important or necessary for knowledge of ethical aspects of risk. She provides several possible interpretations of this claim, some of which she finds less problematic. She argues that as long as emotions concern concrete, immediate circumstances it is plausible to think they are important for moral knowledge. Hayenhjelm finds it more problematic to claim that emotions could play a role in more generic, theoretical and abstract moral knowledge. She thinks that this could be acquired in a purely rational way. Let us look at these arguments in more detail.

Hayenhjelm paraphrases me as saying that emotions are necessary for moral knowledge, and at a certain place she reconstructs my view in an even more radical way:

‘To the extent that *only* moral emotions can “read” the world morally and *only emotions* can track moral reality, emotions are necessary for moral knowledge (and if not, they may still do so in some or most cases).’

However, that is too strong as a generic summary of my theory; rather, whether emotions are sources of moral knowledge depends on the agent and circumstances. I typically use the formulation that moral emotions ‘paradigmatically’ track moral truths. Furthermore, on my view one does not have to be emotional when engaged in abstract, theoretical and generic ethical reasoning; this also requires other capacities, such as logical and conceptual reasoning and processing empirical information. But on the other hand, learning ethics theoretically and merely from a book will probably not provide someone with sufficient resources to make concrete ethical judgments in particular circumstances; this requires moral understanding and context-sensitive insights. For both of these, emotions are needed (cf. Zagzebski 2003; Roeser 2011).

Concerning emotional responses to concrete situations, Hayenhjelm argues that:

‘Risk decisions are seldom about such immediate moral situations. … where any immediate conclusions may be false since they are only part of the picture?’

However, on my view emotions are not only aroused by immediate situations. They also (can, do and should) play a role in deliberation about complex situations, potentially far away.

Hayenhjelm argues that reliability of moral emotions in concrete and abstract deliberation would be different:

“..the reliability does not seem to be the same, given that moral emotions could only react to the situation as presented and thus how an abstract case is described would already determine what is morally salient in some sense…”

To this I would like to reply that the reliability would indeed not be the same but the type of emotional and other reflective capacities would also not be the same. Abstraction could come at a price, by for example an incomplete understanding of a concrete case, or by an insufficient understanding of the impact of events on people because in case of abstract knowledge we are not close enough ‘to the ground’. Nevertheless, I do think that emotions are not always, by definition, reliable, neither in abstract thinking nor in responses to immediate situation. Rather, they are a fallible, but important source of knowledge.

Hayenhjelm takes issue with my claim that “moral emotions in dilemmatic or complex situations are more fallible” (120); she sees that as further confirmation that emotional responses in concrete circumstances are problematic. However, one should note that my observation concerning fallibility of responses to dilemmatic and complex situations would also hold for other, more rational capacities, due to the nature of such cases. Monistic normative ethical theories that take there to be one overarching master principle, such as purist forms of utilitarianism and Kantianism, claim to have straightforward answers to such situations, but more pluralistic and context-sensitive ethical theories argue that this is actually a shortcoming of such monistic theories, as they do not do justice to the complexity of the moral world. On my account, moral emotions are typically triggered by concrete cases, and they are specifically suited to pick up fine-grained morally relevant nuances and to form context-sensitive judgments in particular circumstances. Furthermore, they also can serve as pointers towards genuinely dilemmatic situations, for example when we feel torn between two options.

### Reliability of Emotions and Criteria for Correctness

Hayenhjelm finds it problematic that my approach does not provide a criterion of rightness:

‘…it is hard to see how this would work if there is no objective standard for when something is right or wrong—and without general normative principles and moral intuitions as the ultimate source of moral knowledge, it is hard to see where we would find this.’

On my philosophical approach, the objective standards for when something is right or wrong are constituted by the moral truths that we try to grasp; this is the moral realism aspect of my theory. However, while such a criterion is ontologically mind-independent, once we try to grasp it, we involve our fallible and possible diverging epistemic resources. However, the same holds for traditional, rationalist intuitionism or other moral epistemologies: we need to check our moral beliefs. That’s why in case of disagreement we should deliberate and explore the viewpoint of other people as well. And even if we agree we should always endorse a degree of intellectual humility; we might be bound by group think etc. Actually, cases of agreement could even be more problematic for our epistemic condition than cases of disagreement because that agreement could blind us to our own fallibility.

Based on her reading of my claims, Hayenhjelm argues that ‘moral emotions are both subjective and objective.’ In response, I would say that emotions are subjective and objective in different ways than traditionally conceived. They are personal and fallible experiences and may in that sense be called ‘subjective’, but they are attempts to understand moral truths, and can in that sense be called ‘objective’. So, given that my argument is more modest from the start, by emphasizing fallibility, it is less surprising that I endorse the importance of including a variety of viewpoints. Furthermore, in my view reason is not necessarily ‘more objective’ in the context of moral knowledge (I discuss this in more detail in chapter 5). In my view, the following capacities are needed to gain moral knowledge:

1. Empirical knowledge is needed for insights into descriptive facts, to get the empirical features of the situation right.
2. Logical reasoning is needed for consistent and sound argumentation and for generalizing in terms of patterns.
3. Emotions are needed for grasping moral saliences and for the understanding of the meaning of moral saliences for people, and for generalizing in terms of expanding compassion from near and dear ones to strangers. Note that the latter is especially difficult.

Each of these capacities (1) through (3) is fallible. However, on my view emotions are not by definition more fallible than other epistemic resources. To the contrary, they are needed for moral understanding. Each of the capacities (1) through (3) are verifiable in different ways, requiring appropriate means for their domain. For example, one cannot verify logical reasoning by empirical means, and one cannot check empirical facts by logical reasoning only. The same holds for moral understanding: this requires emotional sensitivity, which cannot be replaced by empirical observations or logical reasoning. Rather, it requires an irreplaceable, genuinely moral perspective, which on my account involves moral emotions.

### The Role of Laypeople

Hayenhjelm discusses in some detail my argument to include laypeople in decision making about risks. She notes that I ‘water down my claims’ by allowing the fallibility of lay emotions. However, as I argued previously, I do make consistently modest claims throughout the book, arguing in terms of importance of emotions rather than necessity. I maintain throughout the book that while taking into account emotions and moral viewpoints of a broad spectrum of people is important, it does not provide for infallible insights. Because of their fallibility they need further scrutiny, involving emotional and other capacities. Emotions should be part of a complex web of deliberation, also involving the capacities I mentioned before, namely, empirical (e.g. scientific) knowledge and logical and abstract reasoning.

Hayenhjelm argues against my account as a reason for lay participation; she argues that democratic arguments and arguments from contractualism are more convincing:

‘since democratic legitimacy could never be achieved without the democratic inclusion of those affected and moral rightness could never be without agreement between those affected (Scanlon 2000).’

My argument does not exclude such arguments. There are democratic, procedural reasons for including lay people, as the ones mentioned by Hayenhjelm. However, I argue that there are also epistemic reasons to include lay people: in order to do justice to a spectrum of viewpoints that can help in a full-blown moral evaluation, as different stakeholders may be alert to different moral saliences which should be included. Furthermore, technocratic and populist approaches typically do not include an explicit reflection on values. These are the approaches I am contrasting my approach with. Democratic decision making should not just be about counting (and maybe manipulating) votes (as in populism) but it should be about deliberation. Hence, we need a rich account of democracy, preferably an account of deliberative democracy, but then expanded with a role for emotions, as I argue in chapter 7. In my book I argue that because laypeople do involve emotions in their responses to risk they also include evaluative considerations about risk that are left out of quantitative approaches, such as justice, fairness and autonomy. Hence, on my view, the democratic and epistemic reasons are both supportive of including laypeople, but given that the epistemic reason is usually not acknowledged I focus on this. Furthermore, in chapter 7 I tie my moral epistemology to deliberative democracy where I explore these interrelationships in more detail.

Against the epistemic argument for including laypeople and their emotions and underlying evaluative concerns, Hayenhjelm argues that:

‘A single, very competent, moral agent with the right kind of emotional and rational capacities could be sufficient to provide the necessary moral input. ‘

I agree: in theory one agent could grasp the moral truths. However, in practice, everyone is in need of fine-tuning, seeing different perspectives etc. Including a variety of people ensures including a variety of viewpoints. Hayenhjelm speculates about a different kind of answer that I might provide:

‘A significant part of Roeser’s overall framework is a commitment to the irreducible pluralism of moral aspects of situations and she would most likely object to the single ideal knower having privileged access to moral truth.’

‘Such an idea could point towards the epistemic superiority of a multitude or a broad set of “knowers,” but does not by itself give a compelling argument for lay inclusions on such grounds.’

To this I would like to reply that we should be careful to distinguish three different types of pluralism:

1. Pluralism of viewpoints (epistemological claim)
2. Pluralism of morally relevant features (ontological claim)
3. Pluralism of moral truths (ontological claim)

I reject (3), for the reason that (3) would be relativistic; I argue against relativism in chapter 3, and I will come back to this in my response to Nyholm [and I provide much lengthier arguments against relativism in Roeser (2005, 2011)]. While I do indeed endorse claims (1) and (2) it is important to note that they are not depending on each other; I endorse them for different types of arguments. My argument for (2) is based on the idea that there is not one overarching moral principle but a plurality of morally relevant considerations that need to be assessed on a case by case basis (cf. Ross 1967/1930, Dancy 2004 and Roeser 2011). Claim (1) is based on what I discussed before: in complex situations, it is hard to get a full grasp of all morally relevant aspects, and consulting different stakeholders can then be very informative. Given the moral complexity of risk decisions, it is epistemically enriching to include such a diversity of perspectives, provided by different stakeholders through their values and emotions. Also, typically experts have a more narrow approach, as they rely primarily on quantitative approaches, and they might miss different perspectives that are needed to assess complex cases.

To summarize my discussion of Hayenhjelm’s worries: on my theory emotions are important, yet fallible gateways to insight into moral truths and understanding of what morally matters. Because of their fallibility as well as because of the moral complexity of decision making about risk, emotions are in need of correction and deliberation, requiring empirical knowledge, sound reasoning but also emotional reflection, which can be facilitated by including a variety of viewpoints and stakeholders.

## Reply to Nyholm

I would like to thank Sven Nyholm for the summary of ideas from my book and for discussing them in detail. His main criticism concerns my commitment to moral realism, which he considers to be problematic as well as unnecessary. Before discussing Nyholm’s arguments in more detail, let me emphasize that I agree that one can adopt my epistemological and normative-ethical claims without following my commitment to moral realism. However, I nevertheless think that moral realism makes my approach stronger, rather than weaker. I will explore this in more detail in what follows, in response to Nyholm’s comments. Before doing so, let me discuss one remark Nyholm makes; he writes:

‘She argues that when we allow our emotions to guide us in our assessments of technological risks, this makes us shrink from narrow, technocratic risk-assessments. Risk assessment should not only be about utilitarian cost–benefit analysis. It should instead take into account a richer range of ethical considerations, like fairness, autonomy, equality, and the like. Roeser argues that if and when we consult our own emotions about risky technologies, most of us will agree with her.’

Here it is important to note that I do not only appeal to introspection but also to, for example, empirical studies from Paul Slovic that show that emotions play an important role in people’s risk perceptions. I argue that Slovic’s empirical findings can be interpreted in a stronger way then he does, based on a cognitive theory of emotions rather than based on Dual Process Theory. Slovic thinks that ‘risk as feeling’ (system 1) can be a heuristic, but needs to be corrected by the more reliable ‘risk as analysis’ (system 2, cf. e.g. Slovic et al. 2004). However, I argue that based on a cognitive theory of emotions, emotional responses to risk can be seen as revealing important moral values that can also be substantiated by ethical theories. Nyholm also writes:

‘it is not a call to always treat all values equally in all situations, but rather a call to take into account the full range of our ethical values in a nuanced and measured way’.

This is indeed my approach, also see my discussion of ethical intuitionism as a pluralistic, context-sensitive normative ethical theory in this context in chapter 3. Most intuitionists reject the idea of there being one general moral master principle from which one could derive ethical judgments in particular cases. Rather, they emphasize that we have to make moral judgments on a case by case basis, taking into account the unique circumstances (Roeser 2011). My own version of ethical intuitionism is what I call ‘affectual intuitionism’, which is a combination of ethical intuitionism with a cognitive theory of emotions (cf. Roeser 2011). In a nutshell, my approach is that ethical intuitions are paradigmatically cognitive moral emotions, i.e. states that are affective and cognitive at the same time, and which provide us with insight into moral values.

### The Perils of Moral Realism

Nyholm argues that a substantive problem of my theory is that it involves a commitment to moral realism, which is a highly controversial theory. He provides quotes by well-known philosophers such as Christine Korsgaard who express their disdain for the type of non-reductive moral realism that I endorse. However, while these are rhetorically nice quotes, they do not contain arguments, which is in the end what a philosophical debate should be about. Also, Nyholm presents these authors as defenders of more modest forms of moral realism. Nyholm writes:

‘On their view, the sort of view that Roeser puts forward is far too controversial and implausible for either realists like them or skeptics about moral realism to be willing to accept it.’

However, it may be a bit far-fetched to call these authors moral realists; there are other moral realists whose views on this matter are close to mine.^1^ Non-reductive moral realists argue that there are belief-independent moral truths, and that this is the most convincing way to avoid ethical relativism. With the discussion in my current book I cannot do justice to the complexity of alternative metaethical views, so in the new book I only give a very short argument and defer the reader to some other sources (by myself and others) where the lengthier discussions are held. In what follows I will respond shortly to the more substantive objections that Nyholm raises based on work by Derek Parfit (2017). Nyholm invokes the following ideas by Parfit:

‘Moral truths, as Parfit puts things, have no “ontologically weighty” implications. (Parfit 2017, 183)’

I do not think that this is in contradiction with anything I say. I guess Parfit means that moral truths do not causally interact with empirical truths, so the wrongness of an action does not change empirical facts. In my view, moral truths supervene on empirical truths. So while changes in empirical facts can give rise to changed moral values, it does not work vice versa. Rather, it would for example require that people change their actions based on moral insights, so there would be a mediation between moral truths and what people do in the light of their understanding of these truths. Nyholm also argues:

‘Unless we use “reality” in some very general sense, reality contains no properties or entities beyond what can be described by the natural and social sciences. … Moral properties and truths do not exist anywhere in space or time, and cannot be perceived.’

I agree that empirically measurable reality can only be described by empirical disciplines. The perceptual analogy that I provide in my book is an analogy; moral perception has parallels but also differences with sense perception. I argue that we can perceive moral truths, but these supervene on empirical facts. In order to perceive the moral truths, we need empirical knowledge, that is, sense perception and/or scientific knowledge, for example measurable facts about a technology and its possible impacts. However, while such empirical knowledge is necessary, it is not sufficient for well-grounded moral knowledge. In order to have moral perception we need emotions to be sensitive to the supervening moral truths.

Nyholm argues that ethical reasoning about concrete cases is based on general principles:

‘..ethical arguments put forward values or ethical principles and relate these to features or aspects of the situation or the problem at hand.’

This claim does not acknowledge the debate in normative ethics concerning top-down versus bottom-up moral reasoning. Ethical intuitionists and other contextualists in ethics argue that moral judgments of concrete circumstances require assessing the specific circumstances, which goes beyond applying general principles, as we need to be aware of how specific features interrelate in the case at hand. Dancy (2004) argues in favor of moral particularism, but also the older intuitionists (e.g. Ewing 1929, Ross 1968/1930, and Broad 1951/1930) have argued that we need to make holistic moral assessments of concrete cases. Nyholm also argues:

‘We accept some propositions about values or moral principles based solely on the inherent plausibility of those propositions—Parfit’s favorite example is that agony is bad—but this does not amount to perceiving any distinctive aspects of reality (Parfit 2017).’

I agree that this can hold concerning generic propositions, but not per se concerning particular moral truths. Furthermore, the intuitionist Ross has argued that we achieve insight into general moral principles via intuitive induction: our knowledge of general moral truths is grounded in particular experiences (Ross 1967/1930). Zagzebski (2003) has argued that insight into general moral principles is based on particular moral judgments, fed by emotions. I would phrase it as follows: that agony is bad is not just a conceptual truth, we fully understand its badness based on emotional experiences (also see my discussion of Steinert’s comments below concerning cultivating emotions).

### Can We do Without Claims to Moral Truth?

Nyholm thinks that we do not need to appeal to moral truths, and he appeals to Scanlon for this:

‘All that it implies is that there is a distinctive type of reasoning we can use to satisfy ourselves that one side of some issue has a stronger argument to back up their case with than the other side does (Scanlon 2016).’

As an intuitionist I would argue that arguments eventually rest on basic ethical beliefs that are foundational, such as that all human beings have equal worth. A racist will deny this and may deploy rational and coherent arguments based on a denial of the principle of equality, but he will have gotten something substantially wrong from the start. If the racist disagrees with the principle of equality we may not be able to persuade him by arguments, but maybe by appealing to compassion and understanding of the inherent humanity of people he initially thought to be less worthy.

Nyholm raises the following point:

‘Saying that one party to a disagreement has less advanced intuitive powers or less well-developed moral perception is not a good and respectful way to try to settle any moral dispute. Nor do I think that Roeser would herself recommend the use of such arguments in ethical deliberations..’

Indeed, I wouldn’t. It is one thing to try to understand the relationship between moral arguments, moral perceptions and moral truths, another how to engage with people with whom we are in a dispute about what we consider to be moral truths. A metaethical approach will not settle this, in the end it will be about our moral perceptions and arguments—but it’s not about just trying to persuade others, whether by rhetoric or arguments, but about trying to find out what is right.

Nyholm writes:

‘It only implies that we have standards for evaluating arguments and methods for formulating moral arguments that we can expect others to take seriously whether they ultimately accept them or not. This might push us in the direction of the sort of light-weight moral realism that Dworkin, Nagel, Scanlon, Parfit, and others are defending.’

I fully accept ideas about the importance of arguments and reasoning, but that’s not sufficient. If I have a coherent scientific argument based on wrong empirical facts, I will not be able to (reliably) derive correct empirical conclusions. Similarly, I need to ground my ethical arguments in a correct understanding of moral truths. Of course, this is not a simple, linear process, even if we may be able to analytically reconstruct it as such. In real life moral deliberation, this is an iterative process, and one that requires, next to argumentative capacities, also emotional and narrative capacities, along the lines that I defend in my moral epistemology: we need emotions in order to understand, assess and evaluate other people’s points of views as well as in order to assess what morally matters.

## Reply to Steinert

Steffen Steinert provides a very nice summary of my book, for which I would like to thank him. He also raises several concerns about my theory, one related to the relation between moral emotions, intuitions and basic beliefs, and one related to how one can cultivate one’s moral emotions. I will discuss these concerns one by one.

### The Relation Between Moral Emotions, Intuitions and Basic Beliefs

Steinert’s first set of concerns partially relates to my ideas about ethical intuitionism. Please note that my detailed account of ethical intuitionism is developed in my previous monograph (Roeser 2011), so in the new monograph I do not discuss all the details of intuitionism, as the focus is primarily on risk. But of course the new book should be self-explanatory, so I appreciate Steinert’s request for further clarification, which I will try to provide in what follows.

Steinert writes:

‘According to Sabine Roeser, and other intuitionists, our ethical intuitions can be understood in analogy to axioms in mathematics: “Ethical intuitionists believe that ethical intuitions function in a similar way as axioms in mathematics in that we cannot argue for them any further, but they can still be taken to be justified” (p. 40).’

Here I would like to caution the reader, as in the section Steinert refers to, I discuss conventional intuitionism, which is rationalistic; in other words: conventional intuitionists argue that ethical insights are objective and that only reason can discover objective truths. My own version of intuitionism, affectual intuitionism, comes later in the book. There I argue that ethical intuitions are paradigmatically cognitive moral emotions. The perception analogy is key in my own version of intuitionism; the analogy with mathematics is more plausible in the context of rationalist versions of intuitionism. Steinert argues:

‘I think that the more fitting analogy is between mathematical axioms and basic moral beliefs.’

However, I identify basic moral beliefs and moral intuitions, so that would not make a difference on my account. It is not clear to me how Steinert distinguishes between these two notions. Steinert also proposes another distinction:

‘I take this to mean that moral emotions serve as intrinsic evidence for basic moral belief, not that moral emotions are basic moral beliefs.’

Based on this, Steinert continues:

‘In a nutshell the clarified picture looks like this: By analogy to axioms our basic moral beliefs can have (1) intrinsic evidence, in the form of ethical intuitions, and (2) extrinsic evidence. Extrinsic evidence here may be something like fit with other basic moral beliefs. Further, these basic moral beliefs can form the basis of complex moral beliefs.’

It is unclear to me what Steinert means by distinguishing these notions. Is the one ontological, the other epistemological? I.e. is the one the axiom/moral truth, the other the belief about it? Or does Steinert mean properly basic versus improperly basic beliefs? Please note that I distinguish between properly basic beliefs, i.e. ones that cannot be justified by other moral beliefs, and improperly basic beliefs, those that can be justified by other beliefs, but also be justified in and of themselves. Steinert asks:

‘Do we have access to basic moral beliefs (or values) *exclusively* via moral emotions or are there other forms of ethical intuition that are non-emotional?’

I say in the book that ethical intuitions are ‘paradigmatically’ cognitive moral emotions. Steinert continues:

‘Then again, for the sake of parsimony we may want to assume that there really is only one kind of ethical intuition.’

I have to confess that I think that parsimony is overrated. Reality is messy and complex, and we need theories that do justice to that complexity. In my view, accuracy is more important than simplicity. So while I acknowledge that there may be purely rational ethical intuitions, I do think that paradigmatically ethical intuitions are cognitive moral emotions. Also, I argue that our general moral beliefs are grounded in concrete experiences (cf. Ross 1939 on intuitive induction) which tend to be more emotional (cf. Zagzebski 2003 on the ‘thinning’ of concrete emotional moral experiences when these get more general and abstract as well as less emotional).

Steinert writes:

‘To summarize, it seems that the analogy between basic moral beliefs and axioms nudges us to think that intuition for basic moral beliefs only takes one form. Basic moral beliefs are supposed to be analogous to axioms and axioms only have one form of intrinsic evidence and that is rational non-emotional intuition.’

To repeat my previous points, there are two analogies, the axiom-analogy and the perception analogy. When I discuss the mathematical, axiomatic analogy I refer to traditional, rationalist intuitionism. When I refer to the perception-analogy, I primarily refer to my own version that gives emotions an important role.

### How to Cultivate Moral Emotions?

Steinert ends his commentary by challenging me to say more about how we can cultivate moral emotions:

‘Given that Sabine Roeser seems to think that emotional sensitivity is important, I would have liked to hear more about the circumstances and frameworks in which emotions can unleash their constructive potential and where emotions are facilitating democracy.’

I think that this is an excellent question, and I would like to thank Steinert for pushing me to say more about this. In the book I address a few ways in which we can critically engage with our emotions: by engaging in ‘emotional moral reflection’, for example by involving meta-emotions, that let us critically reflect on our initial emotions (chapter 6), by emotional deliberation with others, for example in a political context (chapter 7), and by engaging with art works that reflect on and engage with risky technologies (chapter 8). To this latter suggestion Steinert responds by highlighting that involving arts as such may not be a guarantee for fine tuning and cultivating our moral emotions, it also requires the right setting. Indeed, I agree that art is also not an infallible source of emotional-moral reflection. As all sources of insight and reflection, it can be fallible, manipulated and distorted. Cultivating emotions via art requires involving different perspectives, and providing for frameworks, settings and environments through which to critically reflect on artworks as well as on one’s emotional responses. Steinert suggests:

‘Maybe something along the lines of night schools that offer courses on how to improve emotional sensitivity. Another idea is that we make emotional cultivation a part of the regular curriculum.’

I think that emotional cultivation should indeed be part of the regular curriculum. Arguably, emotional cultivation happens in moral education in families and schools, very similar to Aristotelian ideas on habituation. Furthermore, moral education, which on my view includes education of emotions, should be an integral part of the whole curriculum, not just as a separate activity. Given that all aspects of our lives are intrinsically value-laden, be it concerning politics, medicine, economics or technology, moral reflection should be a key part of education in these fields, and that, on my theoretical framework, would also require emotional reflection, habituation and education.

## Conclusion

I would like to thank the three commentators for their extremely thoughtful comments. To summarize, while they all are welcoming certain aspects of my theoretical framework, they think that certain parts of my framework can be dropped. Hayenhjelm thinks that emotions may not be required as they can be replaced by general reasoning, and the public can be included on democratic grounds. Nyholm thinks that my controversial commitment to moral realism unnecessarily weakens my account and can be left out, and Steinert thinks that emotions play a less important role and that ethical intuitions can do a lot of the work. I have tried to show that some arguments of my commentators rest on misunderstandings of my view, while concerning other points we have some more substantive disagreements. Nevertheless, I hope that by clarifying some of my points it also emerges that our disagreements are less substantive than they may have seemed. In short, my view is that emotions are important, yet fallible sources of insight in moral truths, also in the context of risky technologies, and that we need reflection, deliberation and cultivation of emotions in order to let them unleash their valuable potential, rather than being the destructive or at most irrelevant force as which they are often seen.

## Footnotes

1 Korsgaard is not a substantive realist but a procedural realist. Gibbard is a non-cognitivist, which is a substantively different view from moral realism. There are other, influential non-reductive moral realists who defend detailed arguments of their views that are close to my own views on the matter, such as Jonathan Dancy, Terence Cuneo, Russ Shafer-Landau, and David Enoch. Please note that in the current book I only discuss the ontological side of the story very quickly. My more lengthy discussion of moral realism is in my previous monograph (Roeser 2011).
